# Gender and socioeconomic inequalities in changes in a Mediterranean lifestyle among elderly Italians

**DOI:** 10.1093/eurpub/ckac129.413

**Published:** 2022-10-25

**Authors:** M Bonaccio, F Gianfagna, C Stival, A Amerio, C Bosetti, L Cavalieri d'Oro, A Odone, A Zucchi, S Gallus, L Iacoviello

**Affiliations:** 1 Department of Epidemiology and Prevention, IRCCS Neuromed, Pozzilli, Italy; 2 EPIMED, Insubria University, Varese, Italy; 3 Mediterranea Cardiocentro, Naples, Italy; 4 Department of Environmental Health Sciences, IRCCS Mario Negri Institute for Pharmacologic Research, Milan, Italy; 5 DINOGMI, University of Genoa, Genoa, Italy; 6 IRCCS San Martino Polyclinic Hospital, Genoa, Italy; 7 Department of Oncology, IRCCS Mario Negri Institute for Pharmacologic Research, Milan, Italy; 8 Brianza Health Protection Agency, Monza, Italy; 9 Department of Public Health, University of Pavia, Pavia, Italy; 10 School of Medicine, Vita-Salute San Raffaele University, Milan, Italy; 11 Bergamo Health Protection Agency, Bergamo, Italy

## Abstract

The COVID-19 pandemic and the adoption of restrictive measurements to control the SARS-CoV-2 spread disrupted general population lifestyles including dietary behaviours. However, there is poor knowledge on potential socioeconomic and gender disparities in dietary changes. We conducted a telephone-based survey during fall 2020 on a sample of 4,400 participants representative of the population aged 65-99 years living in Lombardy, Italy. Changes in a Mediterranean lifestyle were assessed retrospectively by asking participants to report modifications in the consumption of nine food groups and five diet-related behaviours (e.g., consumption of organic and local foods) compared to the previous year (2019). We then computed a Mediterranean COVID-19 Pandemic Score (MedCovid-19 Score), reflecting changes during pandemic, ranging from -14 to 14, with increasing values indicating improvements in line with a Mediterranean lifestyle. Overall, 18.3% of the study participants worsened their Mediterranean lifestyle (MedCovid-19 Score <0), 35.1% remained stable (MedCovid-19 Score = 0), while 46.6% reported improvements (MedCovid-19 Score ≥1). Predictors of favourable changes toward a Mediterranean lifestyle were educational level (OR = 1.52; 95% CI 1.19-1.95 for postgraduate vs lower education), wealth (OR = 1.52; 1.14-2.02 for high vs low wealth), and skilled manual occupations (OR = 1.57; 1.28-1.92 vs white collars). Women were more likely than men to move away from a Mediterranean lifestyle (OR = 1.86; 1.58-2.21). In conclusion, improvements in line with a Mediterranean lifestyle prevailed in almost half of a large sample of elderly Italians surveyed during the COVID-19 pandemic. However, changes towards a Mediterranean lifestyle were disproportionately distributed across gender and socioeconomic strata. These findings were similar to those from the general population of the Moli-sani study, where it was observed that healthful dietary changes were associated greater wealth.

